# Malabsorption and nutritional balance in the ICU: fecal weight as a biomarker: a prospective observational pilot study

**DOI:** 10.1186/cc10530

**Published:** 2011-11-09

**Authors:** Nicolette J Wierdsma, Job HC Peters, Peter JM Weijs, Martjin B Keur, Armand RJ Girbes, Ad A van Bodegraven, Albertus Beishuizen

**Affiliations:** 1 Department of Nutrition and Dietetics, VU University Medical Center, De Boelelaan 1117, 1081 HV Amsterdam, The Netherlands; 2 Department of Gastroenterology and Hepatology, Red Cross Hospital, Vondellaan 13, 1942 LE Beverwijk, The Netherlands; 3 Department of Intensive Care, VU University Medical Center, De Boelelaan 1117, 1081 HV Amsterdam, The Netherlands; 4 Department of Gastroenterology, Small Bowel Unit, VU University Medical Center, De Boelelaan 1117, 1081 HV Amsterdam, The Netherlands

## Abstract

**Introduction:**

Malabsorption, which is frequently underdiagnosed in critically ill patients, is clinically relevant with regard to nutritional balance and nutritional management. We aimed to validate the diagnostic accuracy of fecal weight as a biomarker for fecal loss and additionally to assess fecal macronutrient contents and intestinal absorption capacity in ICU patients.

**Methods:**

This was an observational pilot study in a tertiary mixed medical-surgical ICU in hemodynamically stable adult ICU patients, without clinically evident gastrointestinal malfunction. Fecal weight (grams/day), fecal energy (by bomb calorimetry in kcal/day), and macronutrient content (fat, protein, and carbohydrate in grams/day) were measured. Diagnostic accuracy expressed in terms of test sensitivity, specificity, positive (PPV) and negative predictive value (NPV), and receiver operator curves (ROCs) were calculated for fecal weight as a marker for energy malabsorption. Malabsorption was *a priori *defined as < 85% intestinal absorption capacity.

**Results:**

Forty-eight patients (63 ± 15 years; 58% men) receiving full enteral feeding were included. A cut-off fecal production of > 350 g/day (that is, diarrhea) was linked to the optimal ROC (0.879), showing a sensitivity and PPV of 80%, respectively. Specificity and NPV were both 96%. Fecal weight (grams/day) and intestinal energy-absorption capacity were inversely correlated (*r *= -0.69; *P *< 0.001). Patients with > 350 g feces/day had a significantly more-negative energy balance compared with patients with < 350 g feces/day (loss of 627 kcal/day versus neutral balance; *P *= 0.012).

**Conclusions:**

A fecal weight > 350 g/day in ICU patients is a biomarker applicable in daily practice, which can act as a surrogate for fecal energy loss and intestinal energy absorption. Daily measurement of fecal weight is a feasible means of monitoring the nutritional status of critically ill patients and, in those identified as having malabsorption, can monitor responses to changes in dietary management.

## Introduction

A persistent negative energy balance, known as protein-energy malnutrition (PEM), depletes lean tissue and adipose mass. In critically ill patients, PEM is strongly correlated with complications, especially infections [1]. In general, malnutrition in critically ill patients is associated with impaired immune function, an increased risk of infections, and an increased mortality rate [2-5]. The latter is linearly related to severity of gastrointestinal failure, as determined by the gastrointestinal failure score (GIF) in ICU patients, which reflects mainly the feasibility of adequate enteral feeding [6].

Patients admitted to an ICU are frequently malnourished and catabolic [7], and therefore, nutrition with adequate amounts of energy and protein, preferably as early as clinically possible via the enteral route, has been strongly recommended [8-11]. It has been demonstrated that it might be difficult to supply the prescribed amount of enteral nutrition in ICU patients, such as in patients with severe hemodynamic failure [12]. Optimal enteral nutrition aims to reduce stress-related metabolic catabolism, to decrease the likelihood of bacterial translocation, and to maintain intestinal mucosal integrity [13]. Because nutritional support does not often meet energy requirements of ICU patients, energy will be derived from body reserves, contributing to the already existing catabolic state [14,15]; adequate and individually customized feeding is believed to be an essential part of treatment of these patients. Both sufficient energy and sufficient protein provision are crucial to ensuring optimal nutrition of this population. Based on current literature, optimal energy needs can be calculated from measured resting energy expenditure (REE) + 10% [16,17]. It has been suggested that the optimal protein supply is 1.2 to 1.5 g/kg pre-illness body weight/day for ICU patients [8,18-20].

Gastrointestinal dysfunction occurs frequently in critically ill patients and is associated with adverse outcomes [21-25]. Because malabsorption may induce and worsen PEM, not only are determining nutritional needs and providing adequate nutritional intake essential, but in addition, nutrient losses have to be considered [26]. Diarrhea, which can be an indicator of malabsorption, is a common feature of ICU patients with multiple organ failure (MOF) and is often neglected in daily ICU practice, resulting in potentially important energy and nutrient losses.

In a former study, we demonstrated that malabsorption is a commonly occurring and neglected clinical problem, contributing to a negative energy balance in one of three ICU patients with diarrhea, and from these data, a fecal weight of > 250 g/day was proposed as a biomarker of malabsorption [27]. This previous small series was extended in the present study to validate fecal weight as a biomarker and to characterize in detail the extent and type of fecal nutrient losses in ICU patients.

Therefore, the aim of the present study is first, to validate the diagnostic accuracy of fecal weight as a marker for fecal energy malabsorption. Second, we aimed to assess fecal macronutrient contents, and subsequently to calculate intestinal absorption capacity of energy and macronutrients in hemodynamically and respiratorily stable ICU patients without known gastrointestinal dysfunction.

## Materials and methods

### Design

This prospective observational pilot study was conducted in a tertiary, mixed medical-surgical academic ICU and focuses on intestinal digestive function. The methods used aimed to measure fecal energy and nutrient losses and subsequently to calculate intestinal absorption capacity from the data obtained. The study extended a previously reported cohort [27].

### Patients

ICU patients had to meet the following criteria for inclusion (a) fully fed with an enteral tube during the previous 2 days, (b) stable hemodynamics and respiratory function according to the treating intensivists, (c) mechanical ventilation with PaO_2_/FiO_2 _ratio > 200 and positive end-expiratory pressure (PEEP) < 15 cm H_2_O, (d) expected length of stay in the ICU three or more days with continuation of enteral nutrition alone, and (e) a stable preadmission weight and no apparent nutritional deficiencies before admission to the ICU. Patients with liver failure, renal failure requiring CRRT (continuous renal replacement therapy), total parenteral nutrition (TPN), and patients with a known intestinal disease or failure (such as stomata, inflammatory bowel disease (IBD), chronic intermittent intestinal pseudooobstruction (CIIP), gastrointestinal tumor, celiac disease, short-bowel syndrome, and so on) were excluded.

Forty-eight consecutive patients were included, and full stool-composition analysis was performed (weight, and amount of energy, fat, protein, and carbohydrates) in 35 patients. In the remaining 13 patients, only fecal weight, energy, and fat content could be assessed (hence, missing values of fecal protein and subsequently calculated carbohydrate content for technical laboratory reasons in the beginning of the study period).

Based on data of the receiver operator curves (ROCs) of stool volume, we divided the patients into two groups (that is, a group with apparent and so-called normal stools (considered not to have malabsorption), and a diarrhea group (with malabsorption).

The Medical Ethics Committee of the VU University Medical Center, Amsterdam, The Netherlands, approved the study protocol, and informed consent by proxy (relatives or contacts) was obtained from the patients.

### Methods

Routinely collected patient data such as APACHE-II score, SOFA score (Sequential Organ Failure Assessment), anthropometric, demographic, and medical data were extracted from the patient data-management system (PDMS; Metavision, Tel Aviv, Israel). Patients' energetic needs were determined by measuring resting energy expenditure (REE) for a minimum of 1.5 hours by indirect calorimetry at baseline [28], by using Datex Deltatrac MBM 100 metabolic monitor (Datex-Engstrom Division, Instrumentation Corp., Helsinki, Finland) after calibration with calibration gas containing 95% O_2 _and 5% CO_2 _before each measurement. The total energy expenditure (TEE) was calculated by adding 10% activity energy expenditure (AEE) to the REE, as recommended in ventilated ICU patients [29].

The actual nutritional intake of energy (TEN), fat, proteins, and carbohydrates was calculated from daily digitalized registration of supplied amounts of enteral nutrition by using gross energetic values (carbohydrate, 4.10 kcal/g; fat, 9.40 kcal/g; and protein, 5.65 kcal/g). These values were adjusted or corrected for urinary nitrogen loss of 1.25 kcal/g, thus reaching a value of 4.4 kcal/g [30,31] per milliliter of feeding. The total amount provided to the patient could thus be calculated by multiplying this by the volume of enteral feeding provided. The no-residue enteral nutrition (Nutrison Standaard/Nutrison Protein Plus/Nutrison Concentrated; Nutricia, Zoetermeer, The Netherlands; or Promote; Abbott Laboratories, Hoofddorp, The Netherlands) was continued during measurements, adjusted according to TEE findings by using our computerized energy/protein algorithm [32], and it subsequently remained unchanged during the study period. This exact amount of delivered feeding was monitored in the PDMS as well.

All feces collections during the study period (72 hours) were carried out by ICU nurses and transferred to preweighed buckets. In the case of watery or loose stools, stool collectors (Flexi-Seal FMS (fecal management system); ConvaTec Woerden) were used; hard stools were collected directly from the hygiene pads. Minimal leakage was accepted, because we defined leakage as none, minimal (a minimal leakage like soiling the sheets), apparent (more than slight soiling of sheets to a definite amount of stools), and completely insufficient collection (for example, loss of sealing of the bag). The score was assessed by the preinstructed ICU nurse responsible for the fecal collections from the patients. Everything more than minimal fecal loss excluded the patient's fecal collection from the study. Feces were weighed (FWW in grams/day), homogenized, and immediately stored at < 4°C until analysis. To measure fecal macronutrient contents and to calculate the intestinal absorption capacity of the ICU patients, feces were analyzed for energy, fat, and nitrogen content. The fecal fat content (F_Fat_) was determined by the Van de Kamer method [33]. On a sample of wet stools, total nitrogen analysis was performed with the micro-Kjeldahl method to determine fecal nitrogen content (F_Nitrogen_) by using previously described catalytic and digestive conditions [34]. Fecal protein content (F_Protein_) was calculated by using a conversion factor, assuming that all of F_Nitrogen _was derived from protein: F_Protein _(g/day) = F_Nitrogen _(g/day) × 6.25. Calories from F_Protein _were calculated by: F_Protein _× 4.4 kcal/day. Subsequently, a sample was taken and freeze dried to be processed by bomb calorimetry, a procedure that determines the heat of combustion of materials that are burned as fuels, and converts this into its energy value [35,36]. These calorimetric determinations represented daily fecal energy loss (F_Energy_) in kcal/day and were performed with the Gallenkamp Ballistic bomb calorimeter, type CBB-33, at University Groningen, The Netherlands. Finally, fecal carbohydrate content (F_Carbohydrate_) was calculated from the nonfat, nonprotein, and nonwater fraction of stools (that is, the fecal "rest", and was calculated with the following formula:

$$\left\{\text{EQ}\right\}{\text{F}}_{\text{Carbohydrate}}\left(\text{g}∕\text{day}\right)\;=\;\left({\text{F}}_{\text{Energy}}-{\text{F}}_{\text{Fat}}\;\times \text{9}.\text{4 }-{\text{F}}_{\text{Protein}}\times \text{4}.\text{4}\right)∕\text{4}.\text{1}0.$$

The intestinal absorption capacity (in percentage) of ingested energy from macronutrients was finally calculated as follows:

$$\left\{\text{EQ}\right\}\left(\text{TEN }-{\text{F}}_{\text{Energy}}∕\text{TEN}\right)\;\times \text{ 1}00.$$

Intestinal energy malabsorption was defined as an absorption capacity of 85% or less [31,37] (derived from intestinal energy-absorption data (90%) in healthy controls; mean minus 1 SD). Specific intestinal malabsorption of fat, protein, and carbohydrate was *a priori *defined as an absorption capacity of 85% or less. Finally, the total energy balance was calculated as

$$\left\{\text{EQ}\right\}\text{TEN }-\text{ TEE }-{\text{F}}_{\text{Energy}}.$$

### Statistical analysis

Descriptive data are presented as means ± SD or medians (with interquartile range) when appropriate. Diagnostic accuracy was depicted by using ROC, and the sensitivity (Se), specificity (Sp), and positive (PPV) and negative predicted values (NPVs) were calculated, as well as the Youden Index (Y, sensitivity + specificity - 1), in which Y = 1 corresponds to a perfect test, and Y = 0 has no diagnostic value [38]. The amount of feces with most optimal diagnostic-accuracy characteristics for malabsorption was used as a cut-off to allocate patients to one of the two groups ("normal stools" and "diarrhea"). Differences between groups were compared with the Student *t *test. The Pearson χ^2 ^test was used to explore relations between variables. The strength of the correlation was determined for continuous variables with the Spearman *r*. An acceptable level of statistical significance was established at *P *< 0.05. All data were analyzed by using software package SPSS (Statistical Package for Social Sciences, Inc., Chicago, IL, USA, for Windows version 15.0).

## Results

Table 1 shows the clinical characteristics of the 48 patients analyzed. As shown, "normal stools" patients and "diarrhea" patients were comparable for sex, age, weight, and height, as well as for energy and macronutrient intake and energy expenditure (Table 2). No difference was found in energy and protein supply of patients between the two groups (29.1 ± 9.2 and 26.3 ± 6.1 kcal/kg/day, and 1.2 ± 0.3 and 1.2 ± 0.4 g protein/kg/day for patients with normal stools and diarrhea, respectively). Use of laxatives, antibiotics, vasoactive medication, and diuretics was not different between both groups as well. Patients did not receive renal replacement therapy as per protocol. APACHE-II and SOFA scores were higher in the group of diarrhea (> 350 g/day) than in the group with normal stools (< 350 g/day), indicative of a more severely ill group.

**Table 1:** ICU patients demographic data per group

	< 350 g/day feces (normal stools)	> 350 g/day feces (diarrhea)	*P *value
Demographic data			
***n***	38	10	
**Gender, ♂/♀**	22:16	6:4	
**Age (year)**	63.1 ± 15.9	64.7 ± 13.8	0.773
**Height**^a ^**(m)**	1.74 ± 0.09	1.70 ± 0.07	0.174
**Weight**^a ^**(kg)**	78.4 ± 16.4	76.1 ± 17.3	0.696
**BMI (kg/m^2^)**	25.9 ± 5.6	26.1 ± 4.3	0.907
**SOFA**^b^	5.6 ± 2.8	9.4 ± 4.1	**0.001**
**APACHE-II**^b^	22.8 ± 7.3	30.6 ± 11.7	**0.023**
**ICU admission**	13 surgical patients	5 surgical patients	
	25 medical patients^c^	5 medical patients	

^a^Height and weight are estimated values. ^b^At day of admission into ICU. ^c^Medical diagnosis included neurologic injury, respiratory failure, sepsis, and others). APACHE-II, Acute Physiology and Chronic Health Evaluation II; BMI, body mass index; SOFA, Sequential Organ Failure Assessment.

**Table 2:** ICU patient energy expenditure and nutrient intake per group

	< 350 g/day feces (normal stools)	> 350 g/day feces (diarrhea)	*P *value
Energy expenditure			
***TEE (kcal/day)^c^***	2,068 ± 380	2,044 ± 361	0.872
Nutrient intake			
**Energy (gross)**			
**(kcal/day)**^d^	2,218 ± 626	1,941 ± 335	0.186
**(kcal/kg/day)**	29.1 ± 9.2	26.3 ± 6.1	0.370
**Fat (g/day)**	77.7 ± 22.4	72.0 ± 16.6	0.457
**Protein (g/day)**	93.1 ± 25.1	85.5 ± 17.9	0.372
**(g/kg/day)**	1.2 ± 0.3	1.2 ± 0.4	0.729
**Carbohydrate (g/day)**	236.9 ± 62.8	216.6 ± 35.8	0.335

^c^TEE, total energy expenditure; *n *= 29. ^d^Energy intake is calculated as gross energy intake, not as energy intake available for metabolization (as is usual with regular calculation of nutrient intake)

### Fecal weight as a diagnostic tool for energy malabsorption

**Figure 1:**
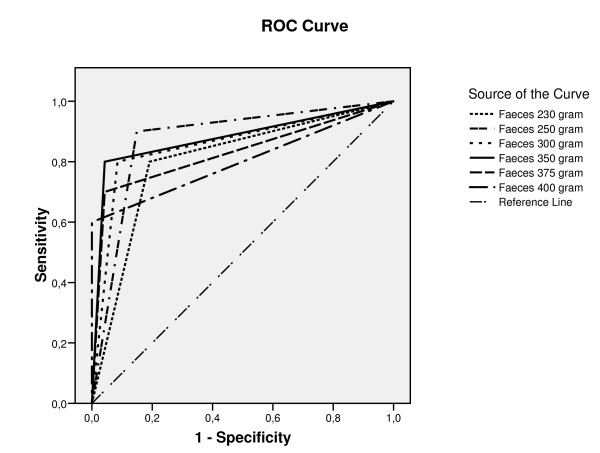
**ROC curves for diagnostic accuracy of fecal weight (grams/day) to diagnose malabsorption in ICU patients**. The receiver operator characteristic curves (ROCs) for diagnostic accuracy of different cut-off points of fecal wet weight (FWW) (ranging from 230 to 400 g/day) as a biomarker for malabsorption. Based on these ROC curves, the diagnostic accuracy of daily fecal weight of 350 g was most optimal, showing sensitivity of 80%; specificity, 96%; positive predictive value (PPV), 80%; and a negative predictive value (NPV) of 96%. Additionally, the accompanying area under the curve (AUC) and the Youden Index indicated that 350 g FWW was the best cut-off value for energy malabsorption.

Figure 1 shows the ROC curves for diagnostic accuracy of different cut-off points of fecal weight (ranging from 230 to 400 g/day) as a biomarker for energy malabsorption. Based on these ROC curves, the diagnostic accuracy (Table 3) was optimal with a cut-off daily fecal weight of 350 g, displaying the largest area under the curve (0.875) with a Youden Index (0.76). Additionally, Se was 80%; Sp, 96%; the PPV, 80%; and the NPV, 96%. The high specificity and high NPV (both 96%) indicated that "no diarrhea" was clinically equivalent to "no malabsorption". If a fecal production of 250 g/day was used as the cut-off, test sensitivity was 90%; thus a fecal weight below this number reasonably excluded malabsorption. However, PPV for actual malabsorption by using this fecal weight as the reference value was only 53%, which may lead to overdiagnosis.

**Table 3:** The diagnostic accuracy of fecal weight in diagnosing malabsorption in ICU patients

Cut-off point (g/day feces)	Sensitivity (%)	Specificity (%)	Positive predictive value (%)	Negative predicted value (%)	Youden index	ROC AUC
**230**	80	81	47	97	0.61	0.804
**250**	90	85	53	98	0.75	0.876
**300**	80	92	67	96	0.72	0.875
350	80	96	80	96	0.76	0.879
**375**	70	96	86	94	0.66	0.829
**400**	60	100	100	92	0.60	0.800

AUC, area under the curve; ROC, receiver operator curve.

**Figure 2:**
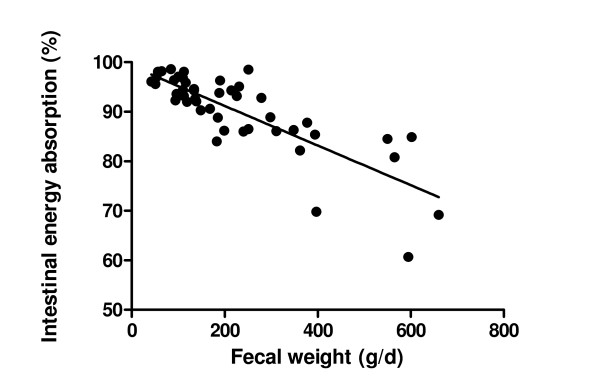
**Relation between fecal wet weight (grams/day) and energy absorption capacity (%)**. The relation between fecal wet weight (FWW) (grams/day) and intestinal energy absorption capacity in 47 stable ICU patients, is inversely correlated (Spearman's *r *= -0.69; *P *< 0.001). More than 350 g/day feces was statistically significantly associated with an energy-absorption coefficient of less than 85% (*P *< 0.001; χ^2 ^test). One outlier is left out of the analysis and picture (3,458 g; 60%).

FWW (g/day) and energy-absorption capacity were inversely correlated (Spearman *r *= -0.69; *P *< 0.001) (Figure 2). More than 350 g/day feces was statistically significantly associated with an energy-absorption coefficient of less than 85% (*P *< 0.001; χ^2 ^test).

### Fecal composition, absorption capacity, and energy balance

Ten (21%) of 48 (*n *= 10) of the included patients were classified as having diarrhea (> 350 g/day feces and were therefore classified to be at increased risk for energy malabsorption. Fecal composition and the intestinal-absorption capacity for energy and macronutrients for both groups are shown in Table 4. Patients with normal stools had a significantly lower total daily fecal energy loss (kcal/d) compared with patients with diarrhea (*P *< 0.001); however, fecal energetic content per gram of feces (kcal/g wet feces) was not significantly different (*P *= 0.135) between groups. Of 48 ICU patients, nine were ultimately diagnosed with energy malabsorption (< 85%), of whom only one patient had a fecal production of < 350 g/day.

**Table 4:** Fecal composition and energy, fat, protein, and carbohydrate absorption capacity for ICU patients with normal stools and with diarrhea

	< 350 g/day feces *n *= 38 (normal stools)	> 350 g/day feces *n *= 10 (diarrhea)	*P *value
Fecal composition			
**FWW (g/day)**	157 ± 79	796 ± 942	**< 0.001**
**Percentage of dry weight (%)**	17.6 ± 5.8	13.5 ± 4.8	**0.047**
**F_Energy _(kcal/day)**	146.4 ± 86.7	445.5 ± 201.3	**< 0.001**
**Energy wet feces (kcal/g)**	0.97 ± 0.36	0.78 ± 0.37	0.135
**Energy dry feces (kcal/g)**	5.49 ± 0.69	5.59 ± 1.09	0.720
**F_Fat _(g/day)**	2.4 ± 2.6	11.4 ± 14.3	**< 0.001**
**F_Nitrogen _(g/day)**	0.9 ± 0.5^a^	2.6 ± 1.1^b^	**< 0.001**
**F_Protein _(g/day)**	5.6 ± 3.1^a^	16.2 ± 7.1^b^	**< 0.001**
**F_Carbohydrate _(g/day)**	24.1 ± 17.7^a^	52.1 ± 18.9^b^	**0.003**
Absorption capacity			
**Energy absorption (%)**	93.1 ± 4.1	76.5 ± 10.6	**< 0.001**
**Fat absorption (%)**	96.8 ± 3.3	84.7 ± 17.2	**< 0.001**
**Protein absorption (%)**	93.6 ± 3.8^a^	82.5 ± 7.2^b^	**< 0.001**
**Carbohydrate absorption (%)**	89.6 ± 6.7^a^	74.5 ± 12.9^b^	**< 0.001**

^a^*n *= 30; ^b^*n *= 5. F_Carbohydrate_, fecal carbohydrate content; F_Energy_, fecal energy loss; F_Fat_, fecal fat content; F_Nitrogen_, fecal nitrogen content; F_Protein_, fecal protein content; FWW, fecal wet weight.

As might be expected, not only F_Fat_, but also F_Protein _and F_Carbohydrate _losses were significantly higher in the patients with diarrhea than in ICU patients with normal stools (*P *< 0.001). As a consequence, energy-absorption capacity was significantly lower in these patients (*P *< 0.001), paralleling fat, protein, and carbohydrate absorption capacity. Protein and carbohydrate absorption capacity were also both negatively correlated with total FWW (Spearman *r *= -0.85; *P *< 0.001; and *r *= -0.69; *P *< 0.001, respectively). The correlation between fat-absorption capacity and FWW was less strong (Spearman *r *= -0.27; *P *< 0.001).

No differences were found between men and women for fecal composition or for intestinal-absorption capacity.

Patients with > 350 g feces/day had a significantly more negative energy balance compared with patients with < 350 g/day feces (loss of 627 kcal/day versus neutral balance; *P *= 0.012).

## Discussion

The current study explored fecal energy and macronutrient composition and fecal weight to see whether this could be used as a diagnostic tool for intestinal absorption capacity in a cohort of stable ICU patients without *a priori *intestinal disease or intestinal failure. In this extended series, we demonstrated fecal weight to be a practical and reliable biomarker for malabsorption in ICU patients; a clinical finding being present in one of five stable ICU patients [27]. A daily fecal weight of more than 350 g provided optimal diagnostic accuracy and may therefore be recommended as a quick and easy screening tool for diagnosing malabsorption in ICU patients.

Nutritional guidelines for ICU patients focus mainly on adequate energy and protein provision. Remarkably, the absorptive capacity of the bowel is not taken into account in any of these guidelines, neglecting the fact that these patients commonly have gastrointestinal dysfunction caused mainly by failing intestinal motility [6]. Malabsorption (here defined as energy intestinal absorption capacity of < 85%, and thus a pragmatic definition of the intestinal function) was frequently observed in this series (one of five stable ICU patients), which even may be an underestimate of the general ICU population, when considering patients in a more-unstable condition, such as sepsis, MODS, and severe trauma. In the patient group with diarrhea, mean daily FWW was approximately 800 g/day, with a mean energetic content of 445 kcal/day, resulting in a mean energetic absorption capacity of 77%, whereas energy and protein absorption in a healthy population is (well) around 90% (data not shown). Malabsorption is an important factor to be considered when aiming to achieve a neutral or preferably positive energy balance in this specific group of ICU patients, as we observed an average negative energy balance of more than 600 kcal/day in the group of ICU patients at risk for malabsorption.

In addition, we demonstrated that in ICU patients with diarrhea (> 350 g/day feces), the observed decreased total intestinal absorption capacity applied for all macronutrients. Daily FWW was inversely related to intestinal malabsorption, constituting a simple biomarker of functional intestinal failure. Conversely, others have shown that postcardiac event ICU patients, without *a priori *intestinal dysfunction similar to our population, had disturbed intestinal motility but a normal intestinal absorption. Apart from a different study method, these patients seemed less severely ill than ours, and the intestinal motility disorders recovered apparently swiftly (only delayed intestinal absorption) [39].

Interestingly, we demonstrated that patients with diarrhea display remarkably higher fecal protein losses compared with patients with normal stools (16.2 g/day versus 5.6 g/day), contributing to the already existing catabolic state. The estimated amount of protein available for the body, calculated as the difference between protein intake and fecal protein loss, decreased below 1.0 g protein/kg/day, which is considerably below the recommended optimal protein needs of 1.2 to 1.5 g protein/kg/day [18-20]. Patients with diarrhea (> 350 g/day) are therefore at higher risk for both a negative energy and a negative protein balance, of which the latter is related to increased risk for infections and mortality [1,2].

The diarrhea frequently observed in ICU may be ascribed to several causes, including the use of drugs or laxatives. Apart from antibiotic use, such as in selective decontamination of the bowel, which is commonly carried out at our IC unit, no medication could be related in any way to the reported findings. Therefore, we suggest that the observed intestinal malabsorption truly reflects a failing organ. Whether this is caused by maldigestion, hypermotility, disbalances of gastrointestinal transport, or other causes cannot be stated with certainty, but is likely mainly to be associated with organ dysfunction of the gastrointestinal tract.

It may be hypothesized that increase of enteral feeding supplies (energy and protein) may have beneficial effects to counteract catabolism in case of voluminous stools in an ICU patient. Whether this should be a form of enteral hyperalimentation using polymeric or semi-elemental formulas in an ICU setting remains to be demonstrated. Finally, one might focus on the benefit versus risks of supplemental parenteral nutrition in case of (unexpected) functional intestinal failure in ICU patients. However, supplying TPN in current clinical practice appears to be reserved for patients with straightforward gastrointestinal failure [40], whereas, interestingly it was recently demonstrated by Thibault *et al *[41] that supplemental parenteral nutrition might prevent a negative energy balance in ICU patients after cardiogenic shock. The timing of starting TPN in the ICU group is under discussion and has recently been studied in the EPaNIC trial; late initiation might be preferable [42].

Several flaws exist in the current pilot study. The number of patients is relatively limited, and measurements were chosen for feasibility in clinical practice to identify a practically applicable biomarker. This limited the precision of measurements of energy and protein losses. On the contrary, the study population had to be fed enterally, which forms the majority (about 90%) of the population of the usual ICU patients. Our studies patients were stable but seriously ill, and *a priori*, no malabsorption was expected. They seemed to be a representative group of ICU patients (for sex, age, height, weight, BMI, SOFA scores, APACHE-II scores, and medical diagnosis), which is a strength of the study. TPN is reserved for a specific small group of ICU patients. The observed findings seem to apply to more-severe patients as well, as long as they are fed enterally.

## Conclusions

In conclusion, we propose to quantify daily FWW in the ICU as a clinically feasible biomarker for clinically significant malabsorption in ICU patients, in particular, in case of diarrhea. Absorption of both energy and all macronutrients is significantly less in patients if fecal output exceeds 350 g/day. Further studies are warranted to confirm the obtained data in this pilot and to establish whether increasing enteral supplies is of clinical benefit to counteract catabolism in these patients. Our findings may improve recognition of potential malabsorption, especially in patients with severe illness and voluminous stools.

## Key messages

• Malabsorption is a clinical finding present in one of five "stable" ICU patients.

• Quantifying daily fecal weight is a clinically feasible biomarker for clinically significant malabsorption in ICU patients.

• Intestinal absorption of energy and macronutrients is significantly lower in ICU patients if the fecal output exceeds 350 g/day.

• ICU patients with a fecal output > 350 g/day are at a high risk for a negative energy and protein balance, with subsequent increased risk for infections and mortality.

• The amount of or the way by which the energy and proteins must be administered must be adapted in ICU patients having voluminous stools.

## Abbreviations

AEE: activity energy expenditure; APACHE II score: Acute Physiology and Chronic Health Evaluation II score; BMI: body mass index; CIIP: chronic intermittent intestinal pseudo obstruction; CRRT: continuous renal replacement therapies; EPaNIC trial: impact of early parenteral nutrition completing enteral nutrition in adult critically ill patients; F_Carbohydrate_: the daily fecal carbohydrate content in grams per day; F_Energy_: the daily fecal energy loss in kcal per day; F_Fat_: the daily fecal fat content in grams per day; FMS: fecal management system; F_Nitrogen_: the daily fecal nitrogen content in grams per day; F_Protein_: the daily fecal protein content in grams per day; FWW: fecal wet weight in grams per day; g/day: grams per day; g/kg: grams per kilogram; GIF: gastrointestinal failure score; IBD: inflammatory bowel disease; ICU: intensive care unit; Kcal/day: kilocalories per day; MOF: multiple organ failure; NPV: negative predictive value; PDMS: patient data-management system; PEEP: positive end-expiratory pressure; PEM: protein-energy malnutrition; PPV: positive predictive value; REE: resting energy expenditure; ROC: receiver operator curve; SD: standard deviation; Se: sensitivity; SOFA score: Sequential Organ Failure Assessment score; Sp: specificity; TEE: total energy expenditure; TEN: total actual nutritional intake of energy; TPN: total parenteral nutrition.

## Competing interests

The author(s) declare that they have no competing interests. The authors have no affiliations or financial involvement with any organization or entity with a financial interest in or financial conflict with the subject matter or materials discussed in the article.

## Authors' contributions

MK included the patients, supervised the collection of specimens, and performed the metabolic measurements. JP and AAB participated in the design of the study and the drafting of the manuscript. PW participated in the design of the study and performed the statistical analysis. NW included the patients, supervised the collection of specimens, performed the metabolic measurements, performed the statistical analyses, and participated in the design of the study and the drafting of the manuscript. AB included the patients, supervised the collection of specimens, performed the metabolic measurements, and participated in the design of the study, and in drafting the manuscript. All authors read and approved the final manuscript for publication.
